# Evaluating the Sub-Acute Toxicity of Formaldehyde Fumes in an In Vitro Human Airway Epithelial Tissue Model

**DOI:** 10.3390/ijms23052593

**Published:** 2022-02-26

**Authors:** Baiping Ren, Qiangen Wu, Levan Muskhelishvili, Kelly Davis, Yiying Wang, Diego Rua, Xuefei Cao

**Affiliations:** 1 Division of Genetic and Molecular Toxicology, National Center for Toxicological Research, US Food and Drug Administration, Jefferson, AR 72079, USA; baiping.ren@fda.hhs.gov (B.R.); yiying.wang@fda.hhs.gov (Y.W.); 2 Division of Biochemical Toxicology, National Center for Toxicological Research, US Food and Drug Administration, Jefferson, AR 72079, USA; qiangen.wu@fda.hhs.gov; 3 Toxicologic Pathology Associates, Jefferson, AR 72079, USA; levan.muskhelishvili@fda.hhs.gov (L.M.); kelly.davis@fda.hhs.gov (K.D.); 4 Division of Biology, Chemistry, and Materials Science, Office of Science and Engineering Laboratories, Center for Devices and Radiological Health, US Food and Drug Administration, Silver Spring, MD 20993, USA; diego.rua@fda.hhs.gov

**Keywords:** formaldehyde, human air–liquid interface (ALI) airway tissue model, fume generation and exposure system, cytokine modulation, squamous differentiation

## Abstract

Formaldehyde (FA) is an irritating, highly reactive aldehyde that is widely regarded as an asthmagen. In addition to its use in industrial applications and being a product of combustion reaction and endogenous metabolism, FDA-regulated products may contain FA or release FA fumes that present toxicity risks for both patients and healthcare workers. Exposure to airborne FA is associated with nasal neoplastic lesions in both animals and humans. It is classified as a Group 1 carcinogen by International Agency for Research on Cancer (IARC) based on the increased incidence of cancer in animals and a known human carcinogen in the Report on Carcinogens by National Toxicology Program (NTP). Herein, we systematically evaluated the tissue responses to FA fumes in an in vitro human air-liquid-interface (ALI) airway tissue model. Cultures were exposed at the air interface to 7.5, 15, and 30 ppm of FA fumes 4 h per day for 5 consecutive days. Exposure to 30 ppm of FA induced sustained oxidative stress, along with functional changes in ciliated and goblet cells as well as possible squamous differentiation. Furthermore, secretion of the proinflammatory cytokines, IL-1β, IL-2, IL-8, GM-CSF, TNF-a and IFN-γ, was induced by repeated exposures to FA fumes. Expression of MMP-1, MMP-3, MMP-7, MMP-10, MMP-12, and MMP-13 was downregulated at the end of the 5-day exposure. Although DNA-damage was not detected by the comet assay, FA exposures downregulated the DNA repair enzymes MGMT and FANCD2, suggesting its possible interference in the DNA repair capacity. Overall, a general concordance was observed between our in vitro responses to FA fume exposures and the reported in vivo toxicity of FA. Our findings provide further evidence supporting the application of the ALI airway system as a potential in vitro alternative for screening and evaluating the respiratory toxicity of inhaled substances.

## 1. Introduction

Airway epithelium is a key interface between the environment and mammalian systemic circulation. Although it serves as an efficient defense mechanism capable of removing inhaled substances upon their entry into the lung [1], the respiratory system represents a major target of environmental toxicants and respiratory pathogens. Evaluating toxicity manifested in this tissue can therefore provide valuable information for assessing the risks of developing respiratory diseases. Despite known discrepancies between the anatomy, dosimetry, and physiology of animals and humans, animal models have traditionally been used as the gold standard for inhalation toxicology. Studies in recent years have further demonstrated that animals fail to fully model the etiology of respiratory diseases in humans [2]. With the enormous number of new chemicals with the potential for human inhalation exposure and ethical concerns about animal testing, inhalation toxicology is facing many unique challenges [3]. There is a pressing need for reliable and predictive models of the human respiratory system capable of efficiently assessing the risks posed by inhaled substances.

To respond to these emerging needs, the National Toxicology Program (NTP) and the National Center for Toxicological Research (NCTR) developed a collaborative project in 2016 to systematically evaluate the value of the primary human air–liquid interface (ALI) airway tissue model for in vitro respiratory toxicity testing. A panel of chemicals with known in vivo respiratory responses, including dihydroxyacetone (DHA) [4], styrene [5], ortho-phthalaldehyde (OPA) [6], glutaraldehyde, and formaldehyde (FA), were selected for testing in this tissue model. Aerosols or fumes were generated from each test article and used for exposing the ALI cultures at the air–liquid interface with the goal of mimicking the inhalation exposures experienced by humans. This report focuses on findings from subacute toxicity testing on FA.

FA is a highly reactive, toxic, and irritating gas that is widely used as an industrial material. It can be generated from diverse sources, including normal metabolism [7], natural processes (e.g., forest fires, microbial products, and plant volatiles) [8], and anthropogenic processes (e.g., motor vehicle exhaust and wood burning stoves) [9,10]. FA also has been used as an antimicrobial agent and monomer or additive in the manufacture of medical device plastics. Overheating polymers can generate FA fumes, which, subsequently, cause an increased rusting of any nearby ferrous parts exposed to the fumes. Due to its widespread presence in the environment and workplace, it is estimated that over 1.5 million people are exposed occupationally to FA in the United States, with approximately 11 million people potentially being exposed in their homes due to off-gassing from building materials and from other indoor air pollutants [11].

Exposure to airborne FA has been linked to both cancer, e.g., respiratory tract cancers [12,13] and lymphohematopoietic cancer [14,15], and noncancer diseases, e.g., sensory irritation [16,17,18,19,20], pulmonary dysfunction [21], asthma [22,23], nasal lesions [24], and congenital malformations [25]. Based on the increased incidence of tumors observed in animals, FA is classified as a Group 1 carcinogen by International Agency for Research on Cancer (IARC) and a known carcinogen by NTP [26,27]. In vitro and in vivo studies have demonstrated that FA produces oxidative damage and is genotoxic [28,29,30,31,32].

Well-differentiated human in vitro ALI airway tissue models, made with primary bronchial epithelial cells, have a complex tissue architecture that retains cellular polarity and the functions found in human airway epithelium [33]. Their ability to maintain their structural and functional properties in cultures for months have enabled the development of assays for assessing disease-relevant subacute tissue responses caused by airborne substance exposures. Previously, we have employed the ALI airway tissue models for elucidating the mode-of-actions of a number of respiratory toxicants, such as acrolein, cadmium, and OPA [6,34,35]. A general concordance for key cellular and tissue responses has been observed between in vitro studies with ALI airway tissue models and in vivo findings.

In the current study, we evaluated whether the major toxic events known to be induced by FA in vivo also are induced by FA exposure of ALI airway cultures. We used a repeated exposure regimen (4 h per day for 5 consecutive days), exposing the ALI cultures to FA fumes at the air interface to better simulate human inhalation exposures. A panel of respiratory disease-related endpoints were assessed at multiple time points to elucidate the temporal airway tissue responses to FA exposures.

## 2. Results

### 2.1. Validation of the Spiking System

**Figure 1:**
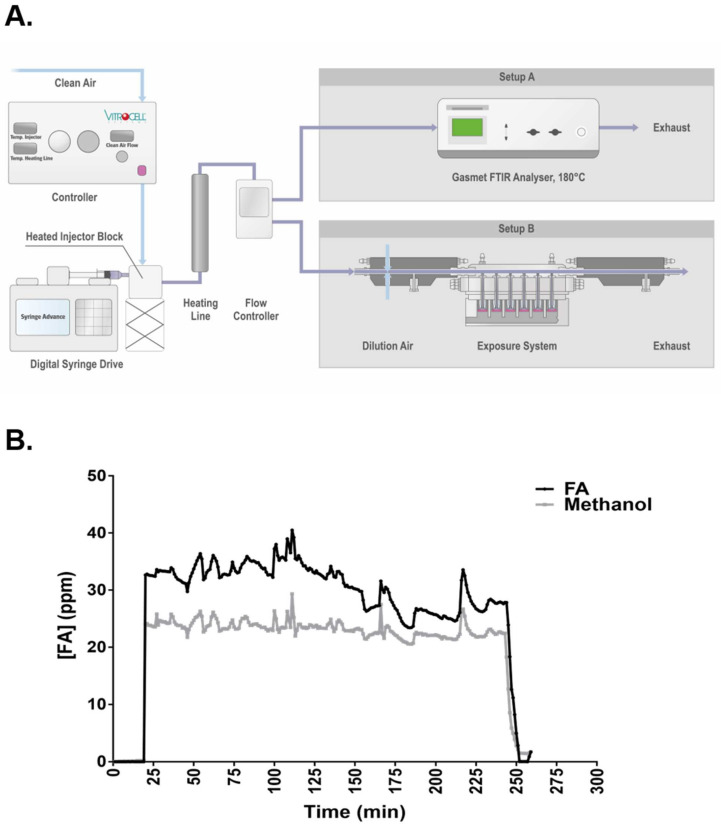
Fume generation and exposure. (**A**) Schematic diagram of the VITROCELL^®^ spiking system. The spiking system consists of a controller, a digital syringe drive, and a heated injector block. The temperatures of the heated injector block and the heating line are controlled by the controller. The flow rate of clean air introduced into the spiking system is also controlled by the controller and was set at a flow rate of 2.0 L/min. FA fumes were directed either to an FTIR for system validation (Setup A) or an exposure system for cell exposure (Setup B) at a flow rate of 0.5 L/min. (**B**) Real-time monitoring of FA fume concentration. Concentration of FA was monitored by an inline FTIR for 4 h. Methanol, which is added as a stabilizer at weight between 10% to 15% in the FA aqueous solution, was also detected in the fumes.

The Spiking System employed for FA fume generation consists of a controller that sets the temperature of the heated injector block and the heating line as well as the air flow rate of the carrier gas (i.e., ultra-zero synthetic air, 85% nitrogen and 15% oxygen; nexAir, Memphis, TN), a heated injector block, and a digital syringe drive (Figure 1A). The concentration of FA fumes was monitored in real-time using an in-line fourier transform infrared spectroscopy (FTIR) over 4 h. In general, the spiking system generated a relatively stable stream of FA fumes throughout the monitoring period (Figure 1B). Setting the temperature of the heated injector block and heating line at 60 °C and the pumping speed of the digital syringe drive at 40 µL/h resulted in a concentration of 30.88 ± 3.81 ppm of FA fumes. Under this concentration of FA fumes, approximately 1.41 ± 0.06 µg of FA was dissolved in the apical liquid layer (or could potentially be taken up by the cells) in the high concentration group over 4 h as estimated by the trapping method. At the same time, 23.26 ± 1.37 ppm of methanol was also detected in the high concentration group of FA fumes. Because of the presence of relatively high levels of methanol in the fumes, its effects on key toxicity endpoints were assessed in a separate experiment and described in the subsequent sections or in the Supplementary Materials.

### 2.2. Cytotoxicity of FA Fumes

**Figure 2:**
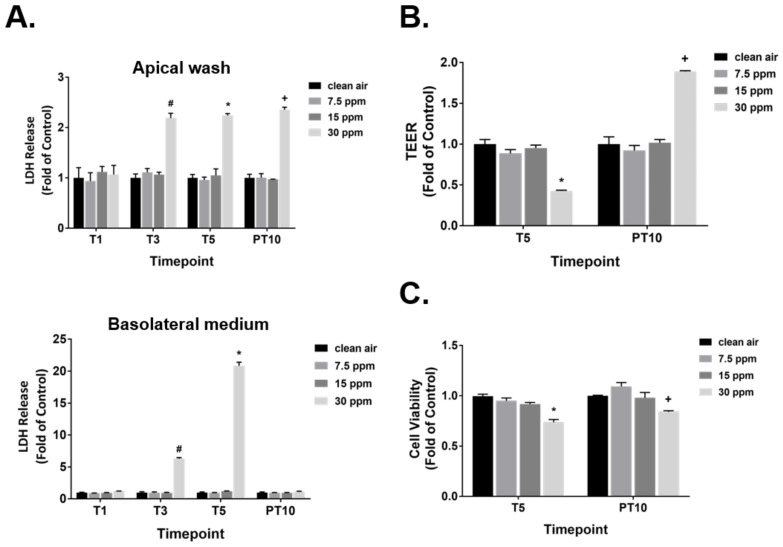
Cytotoxicity of FA fumes in the ALI cultures. (**A**) Release of LDH into both the apical washes and basolateral media was assessed using the LDH assay at T1, T3, T5, and PT10. (**B**) Integrity of the ALI airway tissue was measured at T5 and PT10 using an EVOM2 Epithelial Voltohmmeter. (**C**) Cell viability inhibited by FA fumes was evaluated using an MTS assay at T5 and PT10. Data (*n* = 3) are presented as means ± SEM. ^#,^*^,+^ *p* < 0.05 was considered statistically significant compared to the clean air-exposed controls at T3, T5, and PT10, respectively.

The cytotoxicity of FA fumes in ALI airway cultures was first assessed by measuring the secretion of lactate dehydrogenase (LDH) into both the apical and basolateral compartments at T1, T3, T5, and PT10 (Figure 2A). Release of LDH was not induced by 7.5 and 15 ppm of FA at any of the time points tested. Repeated exposures to 30 ppm of FA, however, significantly increased the secretion of LDH into both the apical and basolateral compartments as early as after 3 repeated exposures. LDH release into the apical side remained at comparable levels between T3 and T5 and was not diminished by a 10-day recovery. LDH release into the basolateral medium, however, followed a different pattern. Secretion of LDH into the basolateral medium increased with increasing numbers of exposures. Despite substantial LDH release into the basolateral side, its level returned to the baseline following a 10-day recovery. Five consecutive daily exposures to 23 ppm methanol had no effect on LDH release in the ALI cultures (Figure S1A).

The tissue barrier properties of the ALI cultures in response to repeated FA fume exposure were assessed by transepithelial electrical resistance (TEER) measurement. Similar to the LDH findings, only 30 ppm of FA fumes caused an approximately 60% reduction in tissue integrity at T5 (Figure 2B). However, following a 10-day recovery, TEER in the high FA concentration group not only increased, but was significantly higher than that of the clean air-exposed control. Five repeated exposures to methanol had marginal effects on TEER (Figure S1B).

Besides cellular and tissue integrity, FA-treated ALI airway cultures were also evaluated for cell viability at T5 and PT10 using the MTS assay. Although significant release of LDH was observed in cultures repeatedly exposed to 30 ppm of FA fumes, the MTS assay, which measures cellular metabolic activity, detected only an approximate 25% decrease in cell viability at T5 (Figure 2C). This decrease in cell viability was partially reversed at PT10 (15% inhibition at PT10 vs. 25% inhibition at T5). Cell viability was not affected by daily exposure to 23 ppm of methanol when measured at T5 (Figure S1C).

### 2.3. Effects of FA Fumes on Apoptosis and Proliferation

**Figure 3:**
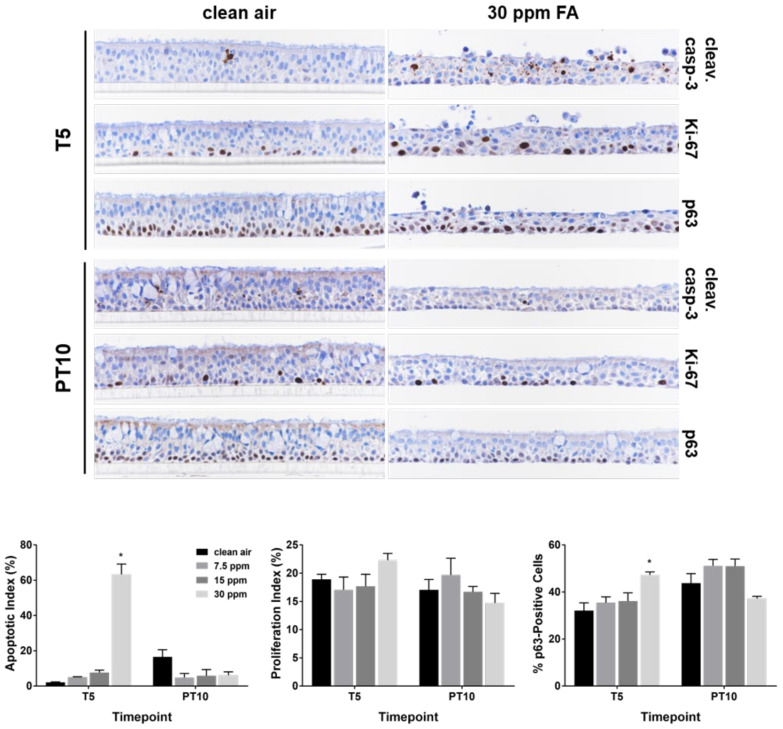
Effects of FA fumes on apoptosis and cell proliferation in ALI cultures. Apoptotic and cell proliferation indices were quantified in tissue sections stained with cleaved caspase-3 and Ki-67 antibodies, respectively. Percentage of basal cells was calculated in p63-immunostained tissue sections. Original images were captured at 40× magnification. Representative images of the clean air- and 30 ppm of FA-exposed cultures are presented (upper panel). Indices of apoptotic, proliferating, or basal cells are shown in the lower panel. Data (*n* = 3) are presented as means ± SEM. * *p* < 0.05 was considered statistically significant compared to the clean air-exposed control at T5.

ALI tissue sections were evaluated with cleaved caspase-3, Ki-67, and p63 antibodies by immunohistochemistry (IHC) to investigate the effects of FA fumes on apoptosis, proliferation, and basal cells, respectively. Repeated daily exposure to FA fumes resulted in increased cleaved caspase-3 immunostaining compared to the concurrent clean air controls (Figure 3, upper panel, T5). Accordingly, apoptotic index (AI) was increased with increasing concentrations of FA fumes after 5 consecutive exposures (Figure 3, bottom panel, T5). The AI in the low and medium concentration groups were higher than that in the clean air-exposed control; however, they were both less than 10%. Five repeated exposures to 30 ppm of FA fumes drastically increased the AI to approximately 60%. After a 10-day recovery, morphological features of apoptosis in the high concentration group disappeared; AI in all treated groups returned to the normal apoptotic levels (Figure 3, PT10).

Staining for Ki-67 and p63 was both primarily localized along the basolateral side (Figure 3, upper panel). Although the proliferation index (i.e., percentage of Ki-67-positive cells) was not affected by repeated exposures to FA fumes, the percentage of p63-positive basal cells was slightly increased by approximately 45% by 30 ppm of FA at T5. Staining for p63 was confined to the nuclei of the basal and parabasal cells and there was a decrease in staining intensity as the distance from the basement membrane increased. The percentage of p63-positive basal cells returned to the baseline level after a 10-day recovery (Figure 3, PT10).

### 2.4. Induction of Oxidative Stress by FA Fumes

**Figure 4:**
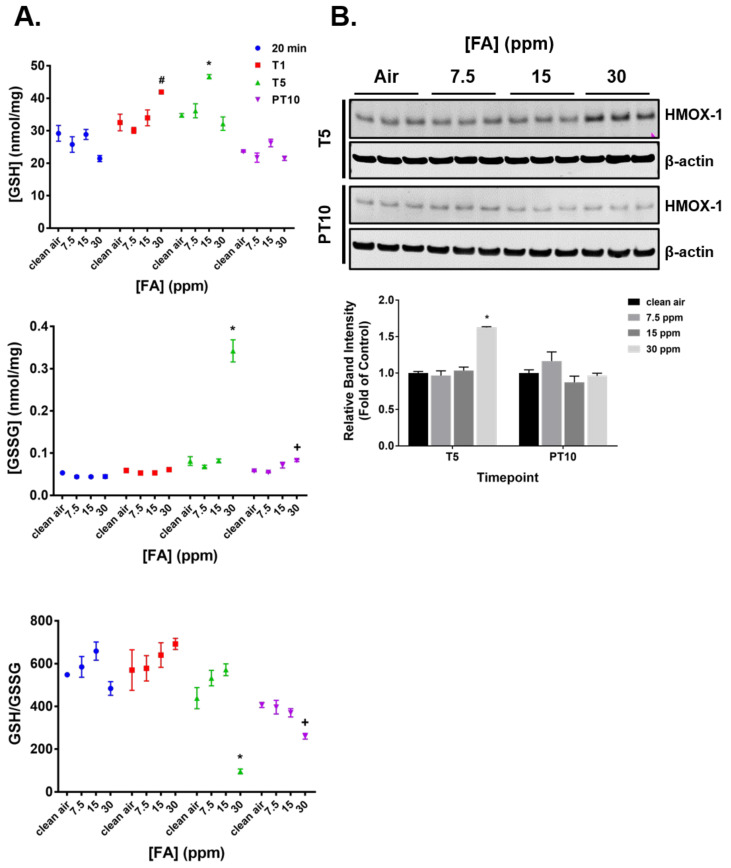
Reversible induction of oxidative stress by FA fumes. (**A**) Intracellular levels of GSH and GSSG were quantified immediately after the first treatment (20 min) and at T1, T5, and PT10. Ratios of GSH/GSSG were calculated at the respective time points and values normalized to the corresponding clean air-exposed controls. (**B**) Protein expression of HMOX-1 regulated by FA fumes was measured at T5 and PT10 by immunoblotting. Representative immunoblots are presented (upper panel). Band intensity of HMOX-1 was normalized to that of β-actin (as loading control) for each sample (lower panel). Relative expression of HMOX-1 was expressed as the ratio of the average band intensity of each treatment group to that of the clean air-exposed control. Quantification data (*n* = 3) are presented as means ± SEM. ^#,^*^,+^ *p* < 0.05 was considered statistically significant compared to the clean air-exposed control at T1, T5, and PT10, respectively.

Oxidative stress was first evaluated by quantifying the intracellular levels of glutathione (GSH) and glutathione disulfide (GSSG) immediately (20 min) and 24 h after the first (T1) and fifth FA exposures (T5), as well as at PT10. A single exposure to FA fumes has no immediate effect on either GSH or GSSH levels (Figure 4A, 20 min). Its effect on GSH homeostasis was delayed and a significant increase in GSH level was observed in the high concentration group after 24 h (Figure 4A, T1). Following 5 consecutive exposures, the GSH level in the medium concentration group was significantly induced, whereas no changes were seen in GSSG levels (Figure 4A, T5). Unexpectedly, repeated exposures to 30 ppm of FA fumes resulted in a substantial increase of GSSG level without apparent modulation of the GSH level. Accordingly, the ratio of GSH to GSSG was significantly decreased in this group. Following 10 days of recovery, the level of GSSG in the high concentration group remained slightly elevated and there was a moderate decrease in the ratio of GSH to GSSG. Repeated daily exposure to 23 ppm of methanol had marginal effects on GSH homeostasis at all time points evaluated (Figure S2A).

FA-mediated oxidative stress was further explored by measuring the expression of HMOX-1 [36] at T5 and PT10. Expression of HMOX-1 was upregulated by 5 repeated exposures to 30 ppm of FA fumes; its expression returned to baseline after a 10-day recovery (Figure 4B). No significant effect on HMOX-1 expression was observed in cultures repeatedly exposed to 23 ppm of methanol (Figure S2B).

### 2.5. Modulation of Ciliary Function and Structure by FA Fumes

**Figure 5:**
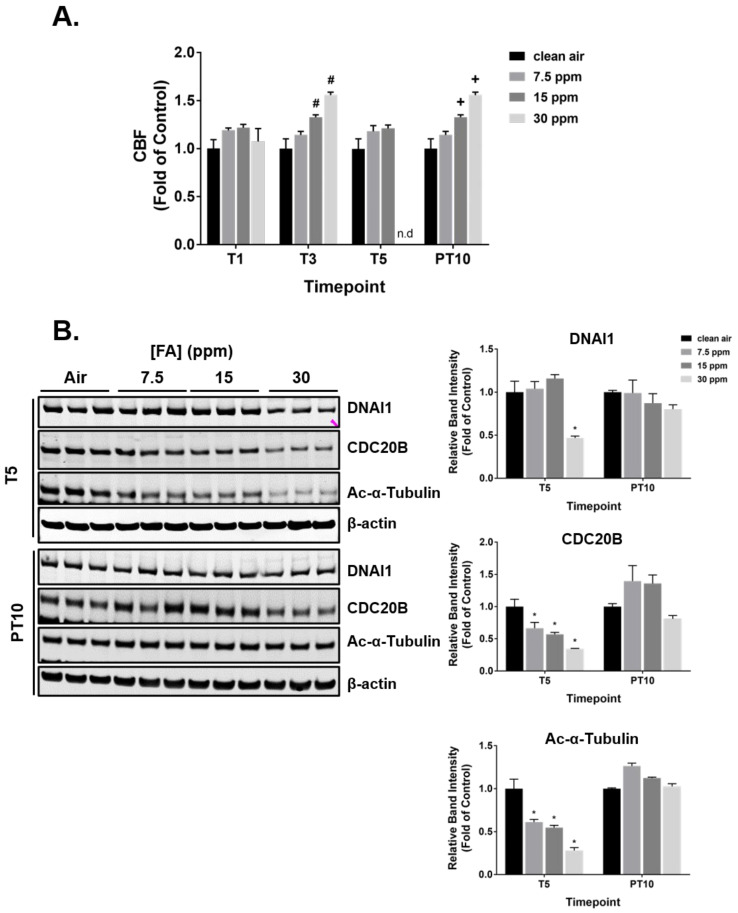
Impairment of ciliary function and structure by FA fumes. (**A**) CBF was measured at T1, T3, T5, and PT10. n.d.: not detected. (**B**) Expression of DNAI1, CDC20B, and acetylated-α-tubulin was measured at T5 and PT10 by immunoblotting. Representative blots are presented in the left panel. Quantification data (*n* = 3) are expressed as means ± SEM. ^#,^*^,+^ *p* < 0.05 was considered statistically significant compared to the clean air-exposed control at T3, T5, and PT10, respectively.

A single exposure to FA fumes had minimal effects on CBF (Figure 5A, T1). Three daily exposures to the medium and high FA concentrations accelerated CBF in a dose-dependent manner (Figure 5A, T3). After 5 repeated exposures, CBF in the medium concentration group was reduced to that in the clean air-exposed control; CBF in the high concentration group, however, was completely abolished (Figure 5A, T5). After a 10-day recovery, the cilia stimulatory effect of FA recurred in a concentration-dependent manner (Figure 5A, PT10). Modulation of CBF was not observed at T5 in cultures exposed to 23 ppm of methanol (Figure S3A).

Expression of select ciliary proteins, i.e., DNAI1, CDC20B, and acetylated α-tubulin, was examined at T5 and PT10 to investigate the potential effect of FA fumes on ciliary ciliogenesis and structure. Repeated daily exposures to 30 ppm of FA fumes downregulated DNAI1 expression; lower FA concentrations had no effect (Figure 5B). In addition, expression of both CDC20B and acetylated α-tubulin was inhibited by FA fumes in a dose-dependent manner. The expression of all three ciliary proteins returned to the baseline at PT10, suggesting the reversible modulation mediated by FA. Methanol had marginal effects on the expression of these proteins (Figure S3B).

### 2.6. Regulation of Mucin Homeostasis by FA Fumes

**Figure 6:**
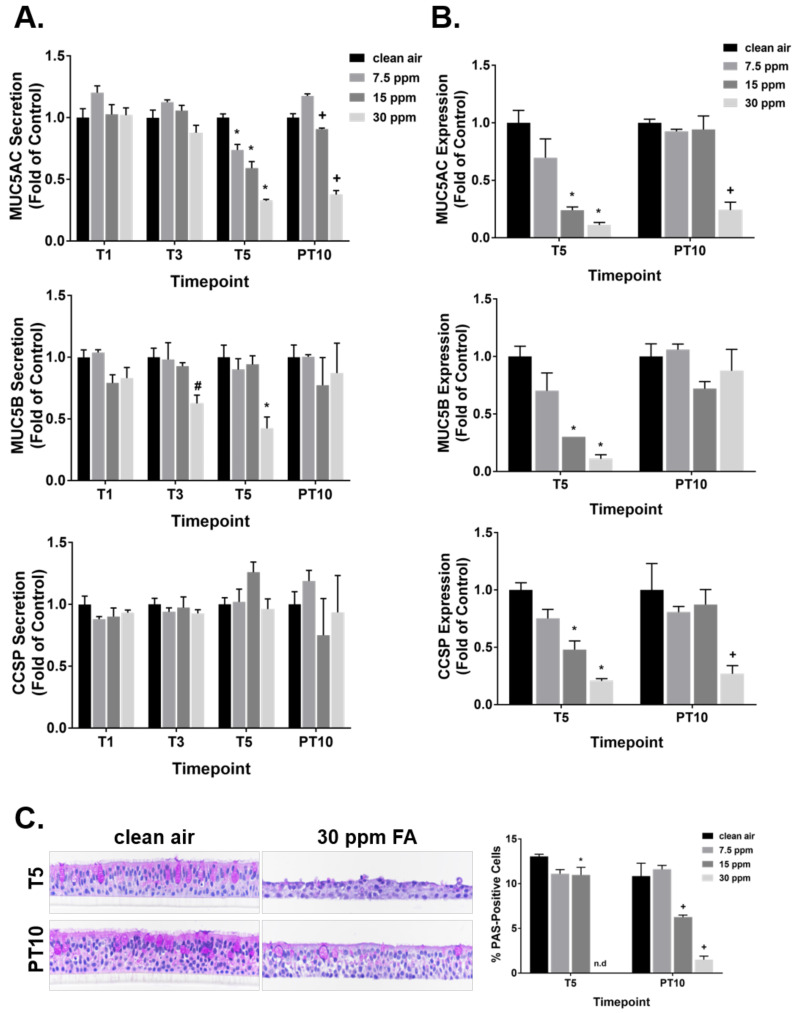
Disturbance of mucin homeostasis by FA fumes. (**A**) Secretion of MUC5AC, MUC5B, and CCSP was quantified at T1, T3, T5, and PT10 using an ELSA assay. (**B**) Expression of MUC5AC, MUC5B, and CCSP was quantified at T5 and PT10. (**C**) Density and morphology of goblet cells were assessed by PAS staining at T5 and PT10. Original pictures were captured at 40× magnification. Percentage of goblet cells was calculated by dividing the number of PAS-positive cells by the total number of the cells in the tissue section. n.d: not detected. Data (*n* = 3) are presented as means ± SEM. ^#,^*^,+^ *p* < 0.05 was considered statistically significant compared to the clean air-exposed control at T3, T5, and PT10, respectively.

The effect of FA on mucin homeostasis was investigated by measuring the secretion and expression of three major airway mucin proteins, i.e., MUC5AC, MUC5B, and CCSP, at T5 and PT10. FA fumes inhibited MUC5AC secretion in a dose-dependent manner after 5 repeated exposures (Figure 6A, top graph). Its inhibitory effect on MUC5AC secretion was sustained following the 10-day recovery. Secretion of MUC5B was decreased only in the high concentration group after 3 repeated exposures (Figure 6A, middle graph). The MUC5B inhibition was reversible and returned to the baseline at PT10. FA fumes had no measurable effect on CCSP secretion (Figure 6A, bottom graph).

With regard to mucin expression, 5 repeated exposures to FA fumes downregulated the intracellular concentrations of all three mucin proteins in a dose-dependent manner (Figure 6B). The inhibitory effect of FA on MUC5B expression was fully reversible at PT10. Expression of MUC5AC and CCSP returned to the baseline in the medium FA concentration group at PT10, whereas their expression in the high concentration group remained depressed following the recovery period. Methanol decreased the secretion of CCSP by approximately 30% only after a single exposure (Figure S4A). Expression of both MUC5AC and CCSP was downregulated by 5 repeated exposures to 23 ppm of methanol. However, the magnitude of inhibition was much smaller compared to the inhibition by 30 ppm of FA fumes (25% vs. 90% for MUC5AC expression and 30% vs. 80% for CCSP expression).

The morphology and density of goblet cells were also assessed by PAS staining at T5 and PT10. Quantification of the PAS staining indicated that 15 ppm of FA significantly decreased goblet cell density after 5 repeated exposures; its negative effect on goblet cell density exacerbated after a 10-day recovery (Figure 6C). Complete loss of goblet cells was observed in the high FA concentration group at T5. After a 10-day recovery, goblet cells re-appeared in this group; their size, however, was smaller compared to that in the concurrent clean air-exposed control.

### 2.7. Downregulation of DNA Repair Enzymes by FA Fumes

**Figure 7:**
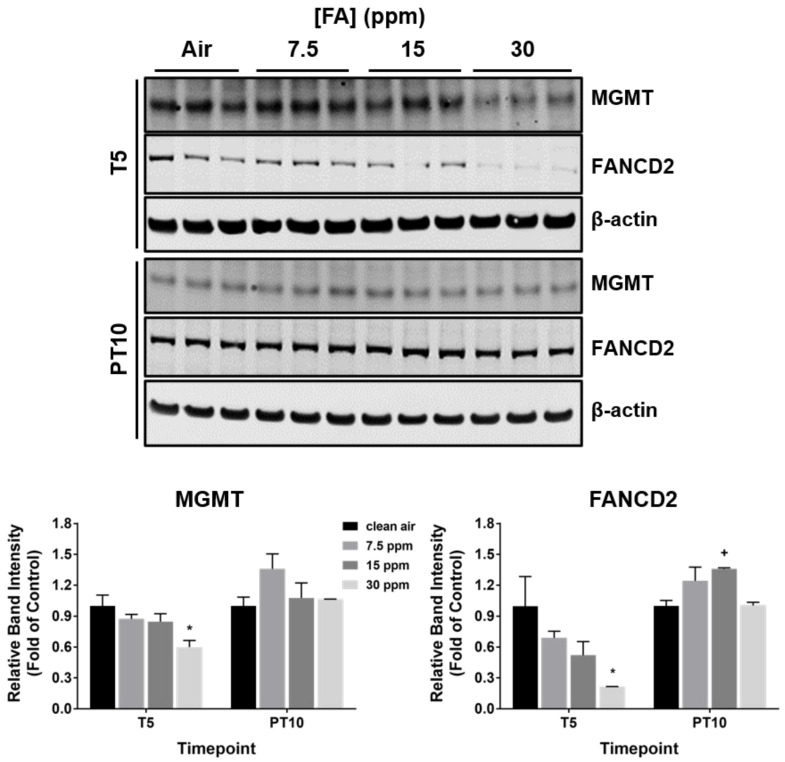
Modulation of DNA repair enzymes by FA fumes. Expression of MGMT and FANCD2 was measured at T5 and PT10 by immunoblotting. Representative blots were presented in the upper panel. Quantification data (*n* = 3) are expressed as means ± SEM. *^,+^ *p* < 0.05 was considered statistically significant compared to the clean air-exposed control at T5 and PT10, respectively.

Previous studies indicate that FA has the potential to both damage DNA and modulate DNA repair pathways [37,38]. We, therefore, examined the expression of two DNA repair enzyme proteins, i.e., O^6^-methylguanine-DNA methyltransferase (MGMT) and Fanconi anemia group D2 (FANCD2), both of which may be involved in FA-mediated DNA damage [39,40], at T5 and PT10. Expression of both proteins was significantly downregulated by 30 ppm of FA fumes after 5 repeated exposures (Figure 7, T5). The inhibitory effect of FA on their expression was generally reversible after a 10-day recovery (Figure 7, PT10). Examination of MGMT expression in methanol-exposed group also revealed downregulation of MGMT, although the magnitude of inhibition by methanol was slightly less than that in the high FA concentration group (Figure S5, 25% inhibition by methanol vs. 40% inhibition by FA fumes). The potential DNA-damaging effect of FA fumes was further explored using the comet assay. The ALI airway cultures were exposed to FA fumes using an exposure regimen reported in the reconstructed human skin comet assay [41]. No changes in % DNA in tail were detected in ALI airway cultures exposed to all three FA concentrations under this experimental condition (data not shown).

### 2.8. Modulation of Inflammatory Molecules by FA

Time-dependent secretion of a panel of cytokines and matrix metallopeptidases (MMPs) was examined at T1, T5, and PT10. In general, repeated exposure to FA fumes resulted in the secretion of pro-inflammatory cytokines. Six cytokines, i.e., IL-1β, IL-1RA, IL-2, IL-8, TNF-α, and IFN-γ, were released into the basolateral medium at significantly higher levels in the high FA concentration group at T5 (Table 1). Release of IL-1RA and IFN-γ in this group was respectively 17- and 12-fold higher than that in the clean air-exposed control. Induction of IL-2, IL-8, and TNF-α was lower, but their concentrations were still approximately 2- to 3-fold higher than in the concurrent clean air-exposed control. Among these cytokines, secretion of IL-1RA, IL-8, and INF-γ was enhanced after the first exposure (T1). Elevation of IL-1β, IL-1RA, and IFN-γ was sustained at PT10. Repeated exposure to the high concentration of FA fumes reduced the secretion of two cytokines, GM-CSF and MCP-1, leading to approximately 2- to 3-fold reductions at T5.

Contrary to its stimulatory effect on all but two pro-inflammatory cytokines, FA inhibited the release of 6 MMPs, i.e., MMP-1, MMP-3, MMP-7, MMP-10, MMP-12, and MMP-13, into the basolateral medium at T5 (Table 1). MMP-7 secretion was inhibited by as much as 70%; MMP-3 and MMP-10 were inhibited by approximately 50%. The effect of FA on MMP-1 and MMP-12 was relatively moderate, with an inhibition of approximately 30%. Among these MMPs, inhibition of MMP-7, MMP-12, and MMP-13, was observed 24 h after the first exposure. Furthermore, MMPs appeared to be more sensitive to FA-mediated inhibition compared to cytokines, as inhibition of select MMP also was detected in the low and medium FA concentration groups. The reduction in MMP secretion was reversible; their secretion returned to the baseline at PT10. The effect of methanol on these cytokines and MMPs generally was marginal (Table S1).

### 2.9. Morphological Changes Induced by FA

**Figure 8:**
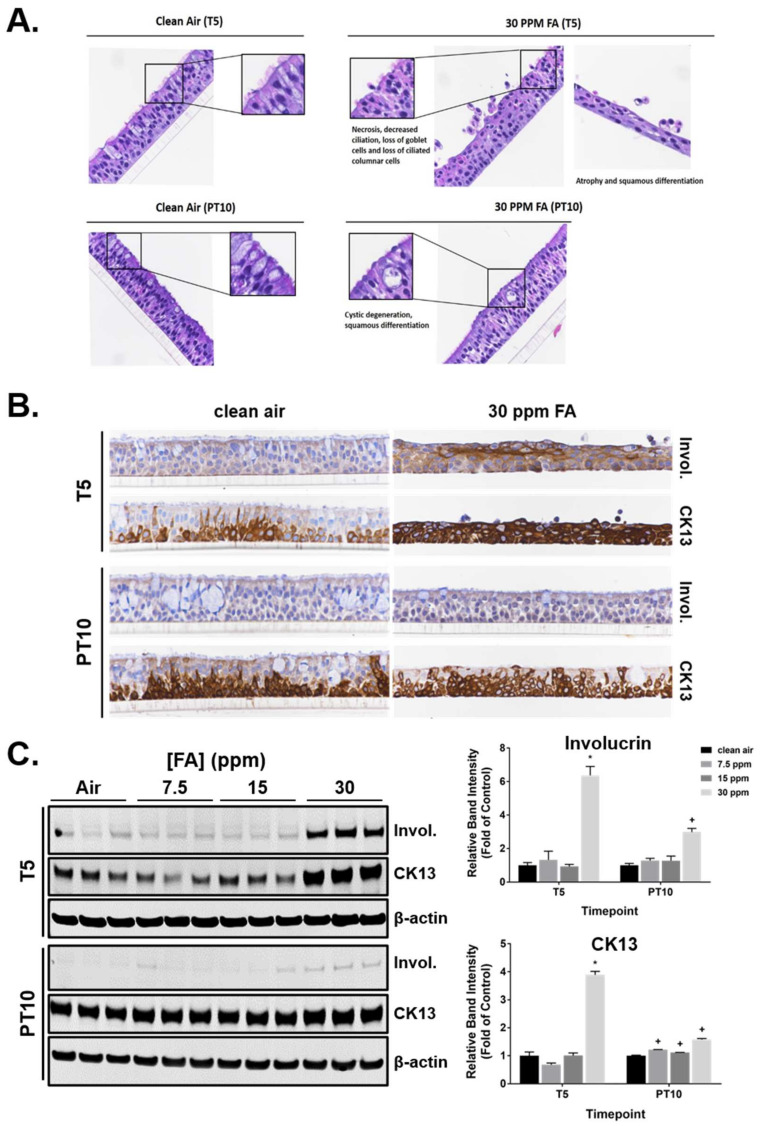
Induction of squamous differentiation by FA fumes. (**A**) General morphological changes induced by FA fumes were assessed at T5 and PT10 by H&E staining. Original images were captured at 40× magnification. Representative areas with key abnormalities are presented in the insets. (**B**) Involucrin and CK13 were detected by IHC at T5 and PT10. Original images were captured at 40× magnification. Representative areas in the clean air- and 30 ppm of FA-exposed cultures are shown. (**C**) Expression of involucrin and CK13 was measured using immunoblotting. Representative blots are presented in the left panel. Quantification data (*n* = 3) are expressed as means ± SEM. *^,+^ *p* < 0.05 was considered statistically significant compared to the clean air-exposed control at T5 and PT10, respectively.

The morphology of the ALI cultures was examined at T5 and PT10 by H&E staining. Multiple morphological abnormalities, including apoptosis, necrosis, atrophy, abnormal cilia, depletion of goblet cells, intra-epithelial cyst, and squamous differentiation, were observed in cultures exposed repeatedly to FA (Table 2). Consistent with the observations of cleaved caspase-3-stained tissue sections, 30 ppm of FA induced moderate apoptosis characterized by shrinkage of single cells, nuclear chromatin condensation, intensely eosinophilic cytoplasm, rounded cell borders with separation from adjacent cells, and fragmentation with budding of cell membranes resulting in small round to oval membrane-bound apoptotic bodies (Figure 8A, inset). Minimal apoptosis was present in clean air-exposed control and cultures exposed to 7.5 and 15 ppm of FA at both T5 and PT10; this was considered normal background unrelated to treatment. Mild to moderate atrophy consisting of focal, multifocal, to diffuse thinning of the airway tissue was observed in cultures exposed to 30 ppm of FA at T5. The affected areas had cuboidal, low columnar cells and/or flattened cells and presented irregular architecture with some loss of tissue organization. Cultures treated with 30 ppm of FA also exhibited minimal to mild necrosis characterized by sloughed individual round epithelial cells on the apical surface at T5.

Decreased ciliation was observed in the medium and high FA concentration groups at T5 and considered treatment related. Cilia abnormalities included an increased number of epithelial cells with no cilia, fewer cilia, and/or cilia of decreased height. The histopathology evaluation corroborated the biochemistry findings on ciliary proteins. Besides ciliated cells, goblet cells were also found to be markedly depleted by 30 ppm of FA at T5. Goblet cell depletion persisted, but was at decreased severity, at PT10.

Importantly, the histopathology evaluation revealed minimal to mild squamous differentiation at or near the apical surface in the medium and high FA concentration groups at both T5 and PT10. Tissues undergoing squamous differentiation consisted of polygonal to spindle-shaped squamoid cells with eosinophilic cytoplasm often containing discernable intercellular bridges (desmosomes). To confirm the histopathology findings, expression of two markers for squamous differentiation, involucrin [42] and CK13 [43], was evaluated at T5 and PT10 using both IHC and immunoblotting. The staining intensities of both proteins group were greatly increased in the FA high concentration group at T5 (Figure 8B). Staining of involucrin tended to be enriched near the apical surface of the cultures; staining of CK13, however, appeared to be dispersed throughout the tissue section. Following the 10-day recovery, the immunostaining intensity of both proteins decreased, yet was still slightly stronger than that in the clean air-exposed control. Immunoblotting revealed a similar pattern in the expression of these two proteins. Repeated exposures to 30 ppm of FA significantly upregulated their expression at T5 and the upregulation was only partially reversed at PT10 (Figure 8C). Repeated exposure to 23 ppm methanol slightly increased the expression of CK13 without reaching statistical significance (Figure S6).

### 2.10. Effect of FA Fumes on Alcohol/Aldehyde Metabolic Enzymes

**Figure 9:**
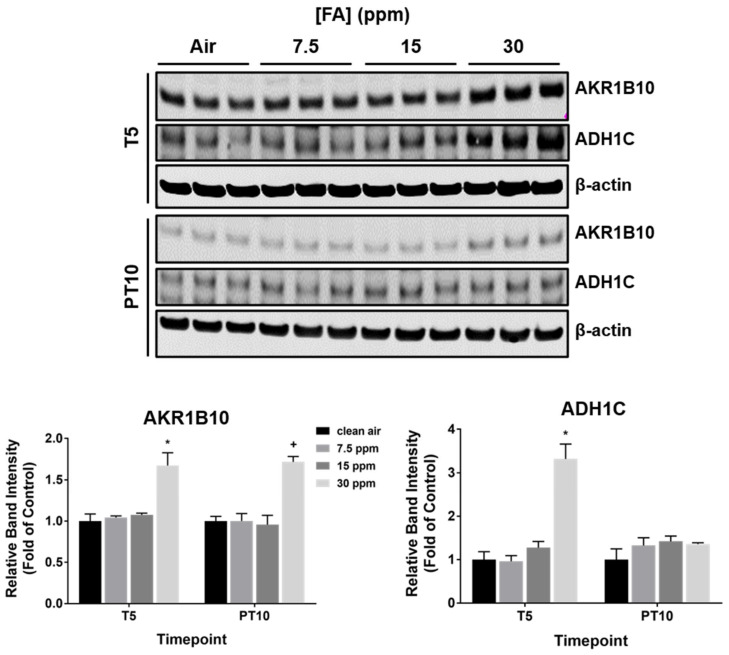
Induction of AKR1B10 and ADH1C by FA fumes. Expression of AKR1B10 and ADH1C was measured at T5 and PT10 by immunoblotting. Representative blots were presented in the upper panel. Quantification data (*n* = 3) are expressed as means ± SEM. *^,+^ *p* < 0.05 was considered statistically significant compared to the clean air-exposed control at T5 and PT10, respectively.

Expression of two alcohol/aldehyde metabolic enzymes, AKR1B10 and ADH1C, was assessed at T5 and PT10. Exposure to 30 ppm of FA greatly increased the expression of both enzymes at T5 (Figure 9). After a 10-day recovery, AKR1B10 remained upregulated in cultures exposed to FA fumes, whereas expression of ADH1C returned to the control levels. Repeated exposures to 23 ppm methanol inhibited the expression of AKR1B10; its effect on ADH1C expression was negligible (Figure S7).

## 3. Discussion

In vitro culture systems ranging from cell lines composed of single cell types to more complex tissue models mimicking the structure and function of respiratory tissues have been used to generate toxicity information on airborne substances. However, none of these in vitro systems has been accepted as a replacement for whole animal inhalation studies [3]. Improvements to in vitro culture models, exposure methods, and assay batteries are urgently needed and comparative in vitro and in vivo data must be generated before adopting any in vitro system as a substitute for in vivo inhalation studies. In the present study, we evaluated the subacute toxicity of FA, a well-known respiratory toxicant with a wealth of in vivo data, using a well-differentiated human ALI airway epithelial tissue model. A spiking system coupled with an in-line FTIR gas concentration monitor was integrated into the test platform and exposures were conducted for 4 h per day on 5 consecutive days. This experimental design was intended to improve the relevance and predictive value of the in vitro findings for in vivo toxicity. Using a reliable method to more accurately estimate exposure to the tissue model, we were able to employ a range of exposure concentrations to achieve minimal, moderate, and strong responses in the endpoints that we evaluated.

The spiking system, manufactured by VITROCELL^®^, is designed for exposing cells at the air interface to gases and fumes generated from volatile and semi-volatile substances [33]. By using the spiking system, Brandwein et al. compared the concentration-dependent toxicity of acrolein administered as either a liquid solution or as fumes and found that the toxicity of acrolein fumes was greater than acrolein dosed as a solution [44]. This study exemplified the value of utilzing fume exposures for toxicity testing of volatile chemicals. Herein, we demonstrated relatively stable generation of FA fumes by the spiking system over the course of 4 h. By using an in-line FTIR, we also found that the fumes generated from the FA aqueous solution used as the starting material also contained approximately 23 ppm methanol fumes along with 30 ppm of FA. The concentration of methanol fumes was higher than was estimated based on its proportion in the FA solution, which likely is due to its greater vaporization efficiency. Although it is present in FA fumes at relatively high concentration, for most endpoints assayed in this study, separate repeated exposures using 23 ppm methanol fumes generated from neat (100% pure) methanol showed marginal responses or responses of much smaller magnitude than the responses produced by the FA/methanol fume mixture. Thus, we conclude that the co-existence of methanol in FA fumes has minimal effects on the responses evaluated in this study.

Exposure to FA causes oxidative stress in rodent airways via multiple mechanisms [30,45,46]. Our obervations revealed that the high FA concentration produced a signficant upregulation in intracellular GSH levels in human ALI airway cultures at T1. The increase in GSH levels suggests that the GSH biosynthesis pathway may have functioned in an adaptive manner to FA exposure [47]. After 5 repeated exposures, the level of GSH in this group was decreased to the baseline level, whereas the GSSG level was markedly increased, resulting in a significant decrease in the GSH/GSSG ratio, a decrease that is indicative of oxidative stress.

As opposed to the high concentration group, the GSH level in the medium FA concentration group was greatly enhanced at T5. This unusual GSH response pattern suggests that additional GSH-dependent mechanisms may have been involved in the response to FA exposure in airway ALI cultures and, thus, confounded the findings on GSH homeostasis. Indeed, FA is known to be metabolized by three alcohol dehydrogenases (ADHs) to either methanol or formate via different intermediates [48], and the ADH1C-mediated metabolic pathway is GSH-dependent. Thus, it is possible that 5 repeated exposures to 30 ppm of FA may have exhausted the newly synthesized GSH that was induced by FA exposures for detoxification catalyzed by ADH1C. This conclusion is corroborated by the upregulation of ADH1C protein expression in the high FA concentration group. As a result, no net change in GSH levels was detected. The medium concentration of FA, however, may have been well within the metabolic capacity of the system. Due to the possible confounding effects of GSH-dependent metabolism, assessment of GSH homeostasis may not represent a reliable method for evaluating the oxidative stress mediated by FA; thus, additional assays are needed. We, therefore, measured HMOX-1 protein expression [36] and protein carbonyl formation [49,50], both of which are robust biomarkers of oxidative stress. The upregulation of both biomarkers at T5 confirmed the induction of reversible oxidative stress by repeated FA exposures.

Defects in mucociliary clearance (MCC) are often observed in respiratory diseases, such as chronic obstructive pulmonary disease and lung cancer [51]. In this study, we indirectly examined the effects of FA exposure on MCC by evaluating responses in mucin homeostasis and ciliated cells. Previous studies indicate that FA produces a bi-phasic response in CBF, where low concentrations of FA accelerate CBF and high concentrations decrease CBF [52,53,54]. Consistent with the literature, three repeated exposures to FA dose-dependently increased CBF in the ALI cultures. After 5 repeated exposures, the CBF in the medium concentration group returned to the baseline while the CBF in the high concentration group was completely abolished. Reduction in CBF was accompanied by the downregulation of proteins involved in ciliary structure and ciliogenesis and either shorter or loss of ciliated cells as revealed by histopathology evaluation, suggesting a causal relationship between ciliostasis and compromised ciliary structures. Although the expression of ciliated proteins returned to the baseline level after a 10-day recovery, the physical effects of FA persisted. In fact, CBF in the medium and high concentration groups was unexpectedly increased at the end of the recovery phase.

FA inhibited both the secretion and expression of three major mucin proteins in human ALI airway cells, accompanied by the loss of goblet cells. This is consistent with the FA-induced mucostasis observed in the isolated frog palate [53]. It is noteworthy that the disturbance of both CBF and mucin homeostasis by FA persisted at the end of the recovery phase, suggesting the potential for long-term adverse effects of FA exposure on MCC.

Overproduction of pro-inflammatory cytokines correlates with the severity and progression of multiple respiratory diseases [55,56,57]. In this study, we found that repeated exposure of human ALI airway cultures to FA fumes induced the secretion of six pro-inflammatory cytokines: IL-1β, IL-1RA, IL-2, IL-8, TNF-α, and IFN-γ. Among these cytokines, the release of IL-1RA and IFN-γ was increased after only one exposure and the increases were sustained throughout the exposure and recovery phases, suggesting their potential roles in FA-mediated inflammatory responses. Consistent with our findings, previous studies have reported that FA exposure increases production of IL-1β, IL-8, and TNF-α in rodents and human lung cells [28,58,59]. Furthermore, human field studies revealed significant increases in TNF-α secretion due to occupational exposure to FA and established a correlation between impaired pulmonary function and cytokine induction in FA-exposed workers [60,61]. Contrary to its inductive effect on cytokine production, FA reversibly inhibited secretion of 6 out of the 8 MMPs that we screened. The role of MMPs in FA-mediated tissue toxicity is not known. We speculate that suppression of MMPs may be a mechanism to either preserve tissue integrity or facilitate thickening of airway walls. Epithelial tissue degeneration and inflammation are believed to be essential mechanisms driving carcinogenesis in FA-exposed subjects [62]. Rodent and human studies have demonstrated that FA exposure results in nasal squamous cell carcinoma [63,64]. In FA-exposed human ALI airway cultures, we found alterations in tissue morphology and upregulation of three squamous differentiation markers, involucrin, CK13, and AKR1B10 [43,65,66], all of which is consistent with squamous differentiation. The increase in TEER observed at PT10 further corroborates the formation of squamous cells in FA-exposed cultures.

This study employed an in vitro respiratory test platform and a panel of disease-related tissue responses to evaluate the toxicity induced by subacute exposures to FA fumes. A full range of responses was observed at the concentrations tested. To confirm the minimal and moderate responses in the low and medium FA concentration groups, the treatment regimen was extended to a total of 4 weeks; however, the nature and magnitude of the responses were essentially unchanged (data not shown). In general, our findings are consistent with observations from a chronic rodent study, which concluded that exposure to higher FA doses for shorter durations causes greater damage than exposure to lower doses for longer durations [67]. Of particular significance, hallmarks of FA-associated respiratory responses, such as oxidative stress, ciliostatsis, mucostasis, pro-inflammatory cytokine induction, and squamous differentiation, are all reproduced in human ALI airway cultures by using the subacute treatment regimen. This demonstrates the concordance in FA toxicity between in vitro and in vivo studies. These findings support the application of the human ALI airway model-based in vitro test platform for toxicity screening or testing of airborne substances.

## 4. Materials and Methods

### 4.1. Fume Generation and Exposure

The VITROCELL^®^ Spiking System (Waldkirch, Germany) was used for continuously generating FA fumes. A 37 wt.% FA aqueous solution (stabilized with 10–15% methanol; Thermo Fisher Scientific, Waltham, MA, USA) was injected into the heated injector block that was pre-warmed to 60 °C at an injection rate of 40 µL/h. For system validation, FA fumes were directed to a FTIR (Gasmet, Vantaa, Finland) at a flow rate of 0.5 L/min (Figure 1A, Setup A). The concentration of FA fumes (and methanol fumes) was monitored in real-time over a duration of 4 h by FITR. For cell exposure, FA fumes were directed to a VITROCELL^®^ 24/48 in vitro exposure system (Figure 1A, Setup B). The FA fumes were serially diluted by ultra-zero synthetic air (herein referred to as clean air; NexAir, Memphis, TN, USA) to 7.5, 15, and 30 ppm within the dilution system and drawn through the exposure system by vacuum at a flow rate of 2.0 mL/min. ALI airway cultures were placed in the exposure module containing PneumaCult™-ALI Maintenance Medium (STEMCELL Technologies, Vancouver, BC, Canada), which were pre-warmed to 37 °C. The apical side of the cultures were exposed to FA fumes 4 h every day for 5 consecutive days, followed by a 10-day recovery period. Clean air-exposed ALI cultures were included as the concurrent vehicle control. A separate set of ALI cultures were exposed to 23 ppm methanol fumes 4 h per day for 5 consecutive days to assess its interference in FA-mediated toxicity.

To estimate the amount of FA dissolved into the aqueous layer covering the cells, stainless-steel inserts (VITROCELL^®^) containing 110 µL of molecular biology-grade H_2_O were placed inside the exposure module and FA fumes (30 ppm) flew through the exposure system for 30 min (hereinafter referred to as the trapping method). Residual H_2_O in the stainless-steel inserts (~85 µL) was collected and FA concentration quantified as described below. The amount of FA trapped in H_2_O (potentially taken up by the cells) over 4 h was estimated by using the following formula.

(1) $$\mathrm{FA}4h\;\left(µg\right)=\frac{\left[\mathrm{FA}\right]\;(µg/\mathrm{mL})\;\times \;85\;µL\;\times \;240\;\min}{30\;\min\times \;1000}$$

[FA] is the concentration of FA measured in ~85 µL H_2_O at the end of a 30-min exposure.

### 4.2. Quantification of FA Using an HPLC-PDA Method

Quantification of FA collected in the stainless-steel inserts was conducted using a high-performance liquid chromatography-photodiode array detection (HPLC-PDA) method. Briefly, FA was derivatized with 2,4-dinitrophenyl-hydrazine (DNPH; Sigma–Aldrich, St. Louis, MO, USA) in phthalate buffer (pH 5.0) to a stable Schiff base product for 2 h at room temperature. The reaction mixture was injected into a Waters Alliance 2695 HPLC system equipped with a 2998 PDA detector (Milford, MA, USA). The FA derivatives were eluted on a Waters Atlantis T3 column (4 × 150 mm, 5 μm) using a mobile phase containing water (Mobile A) and acetonitrile (Mobile B) at a flow rate of 1.0 mL/min. The mobile phase was initially kept at 40% Mobile B, followed by a 4.5-min linear gradient that finished at 100% Mobile B. The composition of the mobile phase was kept at 100% Mobile B for 0.5 min, restored to 40% Mobile B for another 0.5 min, and balanced for additional 4 min. The elution of the derivatization product was monitored at the lambda max of 360 nm. The amount of FA was quantified using a quadratic regression calibration curve ranging from 1.25 to 20 μg/mL with Waters Empower 3 software.

### 4.3. Human ALI Airway Tissue Model

Human ALI airway tissue models were established as reported previously [68]. Briefly, cryopreserved normal human primary tracheobronchial epithelial (NHBE) cells (MatTek, Ashland, MA, USA) were expanded for one additional passage in PneumaCult™-Ex Medium (STEMCELL Technologies) until cells reached approximately 80% confluence. NHBE cells were added onto 24-well PET Transwell^®^ culture inserts on multiwell culture plates at a density of 4 × 10^5^ cells/mL and continued to proliferate in the Ex Medium until they reached 100% confluence. The cells then were washed briefly in Dulbecco’s phosphate-buffered saline (DPBS) and fed from the basolateral side with PneumaCult™-ALI Maintenance Medium for 4 weeks, with medium change occurring every other day.

### 4.4. LDH Activity Assay

The cytotoxicity of FA fumes was evaluated using a LDH Activity Assay Kit (Roche, Indianapolis, IN, USA). Release of LDH into the apical and basolateral compartments was assessed in the apical wash and the basolateral medium, respectively, 24 h after 1 (T1), 3 (T3), and 5 exposures (T5) as well as 10 days after the last exposures (PT10). Apical washes were collected by rinsing the apical surface of the cultures with DPBS (100 µL each time for a total of 2 times). For the LDH assay, 60 µL of the apical wash or 100 µL of the basolateral medium were incubated with 100 µL of a mixture of the kit-supplied Dye and Catalyst Solutions (1/45 *v*/*v*) for 15 min at room temperature in the dark. The reactions were stopped by adding 50 µL Stop Solution to each well. Absorbance at 450 nm was measured using a Synergy H4 microplate reader (BioTek Instruments, Winooski, VT, USA).

### 4.5. MTS Cell Viability Assay

Reduction in cell viability by FA was evaluated using the CellTiter 96^®^ AQueous non-radioactive cell proliferation assay (MTS; Promega, Madison, WI, USA). Cell viability was assessed at T5 and PT10. Cultures were washed briefly with DPBS and then incubated in the MTS/PMS working solution (100 µL, stock solution diluted 1:5 with maintenance medium) from the apical side at 37 °C for 1 h in the dark. Absorbance of the working solution (90 µL) at 490 nm was measured using a BioTek Synergy H4 microplate reader.

### 4.6. Histology and IHC

Histopathologic changes by FA were assessed at T5 and PT10. Tissue block preparation and histopathologic stains were conducted as reported previously [6] with minor modifications described below. For detection of involucrin, the antigen was retrieved in 1 mg/mL trypsin for 5 min at 37 °C. The immunohistochemical staining procedures after antigen retrieval were performed on an Autostainer 360 (ThermoFisher Scientific, Waltham, MA, USA) by going through the same steps as employed by manual staining. Stained sections were scanned and digital images obtained with the Aperio Scanscope System (Leica Biosystems, Vista, CA, USA). The percentages of Ki-67- or p63-positive nuclei were evaluated with the nuclear algorithm (Aperio Scanscope System). This algorithm counts the numbers of positive (brown) and negative (blue) nuclei present in the tissue section. The percentages of cleaved caspase-3-positive apoptotic bodies or periodic acid–Schiff (PAS)-stained goblet cells were evaluated semi-automatically, i.e., the total numbers of nuclei were counted with the nuclear algorithm, while the apoptotic bodies or PAS-stained goblet cells were counted manually.

### 4.7. TEER

The effect of FA on tissue barrier integrity was evaluated at T5 and PT10 by measuring changes in TEER using an EVOM2 epithelial volt-ohmmeter fitted with a STX2 chopstick electrode (World Precision Instruments, Sarasota, FL). Prior to the measurement, cultures were equilibrated to room temperature and the EVOM2 was calibrated to 1000 Ω using a test electrode. DPBS was added to both the apical (200 µL) and basolateral sides (400 µL). Three measurements spaced at 120° from each other were made for each culture and the average was calculated for data analysis.

### 4.8. Intracellular Reduced (GSH) and Oxidized Glutathione (GSSG) Levels

Intracellular GSH and GSSG levels were quantified by following a modified liquid chromatography-mass spectrometry (LC-MS) method as described previously [34]. Briefly, cells were lysed in ammonium bicarbonate buffer containing 10 mM 2-iodoacetamide (IAM) (pH 10.0) by 3 cycles of freeze at −80 °C (5 min) and thaw at 37 °C (1 min). The cell lysate was collected by centrifugation at 12,000× *g* for 10 min at 4 °C. The protein lysate (20 µg) was diluted in 40 µL of 10 mM of IAM buffer and deproteinized with 160 µL acetonitrile containing 0.1% formic acid. Concentrations of GSH and GSSG were quantified using a Waters e2695 Alliance HPLC System coupled with a Waters ACQUITY QDa Mass Detector (Milford, MA, USA) and the elution conditions reported previously.

### 4.9. Immunoblotting

Immunoblotting was conducted as described previously [34]. Briefly, cell lysates extracted using the Pierce M-PER Mammalian Cell Lysis Buffer supplemented with 1% SIGMA*FAST*^™^ Protease Inhibitor Cocktail (Sigma-Aldrich, St. Louis, MO, USA) were denatured, separated on a 4–12% NuPage^®^ Novex^®^ Bis-Tris gradient gel (Life Technologies, Carlsbad, CA, USA) at 200 V for 45 min, and transferred onto a nitrocellulose membrane (LI-COR, Lincoln, NE, USA) at 30 V for 1 h. Proteins were detected by incubating first with primary antibodies (rabbit anti-HMOX-1 antibody, Cell Signaling, Danvers, MA, USA; mouse anti-acetylated α-tubulin, mouse anti-AKR1B10, rabbit-anti-DNAI1, and mouse anti-CDC20B, Sigma–Aldrich; mouse anti-CK13 and goat anti-MGMT, Santa Cruz, Dallas, TX, USA; mouse anti-involucrin, Thermo Fisher Scientific, Waltham, MA, USA; rabbit anti-ADH1C and rabbit anti-FANCD2, Abcam, Cambridge, MA, USA), followed by a 30-min incubation with the IRDye-conjugated secondary antibodies (LI-COR). Images were taken using an Odyssey^®^ CLx Imaging System (LI-COR). Densitometry was conducted using LI-COR Image Studio software.

### 4.10. Cilia Beating Frequency (CBF)

Cultures were equilibrated to 30 °C for 15 min on a heated stage. Videos of cilia motility were recorded using a high-speed digital camera (Ammons Engineering, Mount Morris, MI, USA) connected to a Leica DMI-4000B microscope (Leica Microsystems, Buffalo Grove, IL, USA). Four random fields devoid of mucus clumps were captured for each culture. CBF was quantified using the Sisson-Ammons Video Analysis system (version 2.0.8W, SAVA System; Ammons Engineering).

### 4.11. Mucin ELISA

Secretion and expression of MUC5AC, MUC5B, and club-cell secretory protein (CCSP) were measured in apical washes and cell lysates, respectively, using an ELISA method as described previously [34]. Apical washes (50 µL) or protein lysates (5 µg) were coated onto a NUNC-Immuno^®^ MaxiSorp™ ELISA plate (Thermo Fisher Scientific) at 37 °C overnight. Mucin proteins were detected by incubating for 90 min at room temperature with antibodies against MUC5AC (Pierce), MUC5B (Abcam), or CCSP (Millipore, Burlington, MA, USA), all of which were diluted at 1:500 in DPBS containing 10 mg/mL bovine serum albumin (BSA), 0.3% Triton X-100, and 0.2% Tween-20, followed by a 60-min incubation with HRP-conjugated goat-anti-mouse antibody (Rockland, Limerick, PA, USA) diluted at 1:1000 in the same buffer. Plates were washed three times with DPBS. Color was developed by adding 50 µL 3′,5′-tetramethylbenzidine (TMB, Thermo Fisher Scientific, USA) to each well and the reactions terminated with 50 µL Stop Solution (Abcam). Absorbance at 450 nm was measured using a Synergy H4 microplate reader.

### 4.12. Secretion of Cytokines and Matrix Metalloproteinases (MMPs)

Secretion of cytokines and MMPs into the basolateral medium was quantified using a Bio-plex Pro Human cytokine 27-plex assay kit and a Human MMP 9-plex assay kit, respectively (Bio-Rad, Hercules, CA, USA), by following the manufacturer’s recommendations. Basolateral media (50 µL) and analyte standards were incubated with fluorescent magnetic beads for 1 h with shaking at 850 ± 50 rpm. Following three washes with kit-supplied wash buffer, the beads were incubated with detection antibodies for 30 min with shaking at 850 ± 50 rpm. The protein-antibody conjugates were then mixed with streptavidin (SA)-PE and shaken for 10 min at 850 ± 50 rpm. SA-PE-labeled beads were resuspended in 125 µL Assay Buffer. Fluorescence of the beads was measured using a MAGPIX system (Luminex, Austin, TX, USA).

### 4.13. Statistical Analysis

The data are presented as means ± standard error of the mean (SEM). Statistical analysis was conducted using GraphPad Prism (version 7.04, La Jolla, CA, USA). Data at each time point were analyzed using one-way ANOVA, followed by Dunnett’s test for identifying treatment-related responses.

## Figures and Tables

**Table 1:** Modulation of inflammation-associated proteins by FA fumes.

	T1				T5				PT10			
Analytes (pg/mL)	Clean Air	7.5 ppm	15 ppm	30 ppm	Clean Air	7.5 ppm	15 ppm	30 ppm	Clean Air	7.5 ppm	15 ppm	30 ppm
IL-1β	0.65 (0.01)	0.63 (0.03)	0.63 (0.04)	0.54 (0.00)	0.20 (0.02)	0.24 (0.03)	0.20 (0.05)	0.35 * (0.03)	0.18 (0.01)	0.27 (0.03)	0.20 (0.05)	0.34 * (0.01)
IL-1RA	90.60 (0.34)	81.54 (8.03)	152.53 (17.07)	502.69 * (42.02)	121.14 (4.15)	108.72 (11.02)	178.33 (13.83)	1955.10 * (74.15)	443.82 (34.87)	504.32 (30.97)	544.21 (48.79)	1110.34 * (101.60)
IL-2	7.83 (0.43)	7.97 (0.38)	8.05 (0.21)	7.33 (0.40)	1.97 (0.16)	2.37 (0.13)	2.09 (0.35)	4.00 * (0.24)	6.22 (0.65)	6.09 (0.00)	6.09 (0.36)	7.61 (0.76)
IL-8	6951.04 (52.65)	9657.0 (478.00)	5639.09 (106.83)	18,580.22 * (1710.48)	9714.56 (610.62)	10,345.46 (283.79)	7285.13 (347.27)	27,535.83 * (3104.71)	18,672.19 (761.49)	15,104.70 (1594.71)	20,777.15 (3833.76)	121,615.77 (687.36)
TNF-α	110.91 (7.59)	116.34 (3.95)	85.61 * (1.49)	109.71 (3.76)	95.19 (4.37)	94.58 (3.03)	63.90 * (1.29)	204.11 * (9.53)	178.80 (2.37)	139.51 (10.25)	164.94 (33.25)	139.58 (4.50)
IFN-γ	3.76 (0.12)	3.76 (0.12)	5.94 (0.74)	14.84 * (1.66)	3.94 (0.39)	2.80 (0.10)	5.05 (0.65)	47.51 * (2.60)	13.37 (1.27)	15.33 (1.24)	16.55 (0.88)	28.64 * (2.55)
GM-SCF	9.64 (0.58)	10.59 (0.53	8.41 (0.72)	7.10 * (0.48)	11.32 (0.67)	11.24 (0.50)	7.57 * (0.62)	6.92 * (0.98)	19.94 (0.37)	12.23 (1.83)	17.58 (3.64)	11.96 (0.61)
MCP-1	49.76 (9.04)	37.91 (3.11)	46.31 (4.98)	40.64 (3.41)	64.90 (13.81)	40.41 (3.55)	40.21 (3.18)	19.69 * (2.22)	92.11 (4.01)	124.61 (22.96)	93.59 (22.33)	81.31 (7.93)
MMP-1	35.00 (2.31)	34.17 (0.83)	30.83 (0.60)	32.67 (0.33)	36.75 (1.03)	33.75 * (0.25)	30.25 * (0.48)	24.25 * (1.03)	43.33 (1.20)	42.33 (2.19)	45.83 (3.94)	37.67 (1.77)
MMP-3	28.00 (1.00)	27.67 (0.33)	24.67 * (0.66)	27.00 (0.58)	29.75 (0.75)	26.75 (0.25)	24.00 * (0.41)	18.50 * (1.33)	36.00 (0.58)	35.67 (1.85)	35.67 (1.85)	31.17 (1.74)
MMP-7	8033.83 (1230.46)	6607.83 (554.30)	2111.83 * (266.46)	3945.50 * (205.05)	9820.13 (1190.18)	7314.63 (431.24)	2605.25 * (149.81)	2919.25 * (551.42)	12,142.33 (416.75)	10,102.17 (2002.97)	10,726.67 (2870.16)	7788.00 (2214.15)
MMP-10	15,951.17 (139.48)	15,574.17 (337.10)	15,521.83 (65.21)	15,890.33 (131.23)	15,667.25 (116.16)	15,432.75 (268.67)	15,217.6 (158.37)	9704.00 * (794.19)	16,185.83 (30.51)	15,935.00 (238.98)	16,357.00 (121.24)	15,744.00 (150.73)
MMP-12	143.00 (21.79)	88.00 * (2.08)	56.33 * (2.73)	73.17 * (4.87)	209.75 (28.96)	111.50 * (8.86)	78.00 * (6.48)	84.00 * (8.24)	245.67 (16.75)	297.67 (47.69)	208.67 (31.38)	187.83 (21.90)
MMP-13	5012.50 (216.88)	5163.00 (69.11)	4860.83 (43.80)	5664.17 * (99.18)	5182.38 (160.17)	4764.50 (89.45)	4563.25 (80.21)	3755.00 * (274.16)	6529.67 (152.84)	6969.33 (237.39)	6805.33 (179.84)	6330.50 (105.86)

* *p* < 0.05 compared to the clean air-exposed control; data are expressed as mean (SEM).

**Table 2:** Morphological changes caused by FA fumes.

	T5				PT10			
Microscopic Findings	Clean Air	7.5 ppm	15 ppm	30 ppm	Clean Air	7.5 ppm	15 ppm	30 ppm
Apoptosis	3/3 (1.0) [100%]	3/3 (1.0) [100%]	3/3 (1.0) [100%]	3/3 (3.0) [100%]	3/3 (1.0) [100%]	3/3 (1.0) [100%]	3/3 (1.0) [100%]	3/3 (1.0) [100%]
Atrophy	0/3 (0.0) [0%]	0/3 (0.0) [0%]	0/3 (0.0) [0%]	3/3 (2.7) [100%]	2/3 (1.0) [67%]	2/3 (1.0) [67%]	3/3 (1.0) [100%]	2/3 (1.0) [67%]
Ciliation, decreased	0/3 N/A [0%]	0/3 N/A [0%]	1/3 N/A [33%]	3/3 N/A [100%]	2/3 NA [67%]	2/3 NA [67%]	3/3 NA [100%]	3/3 NA [100%]
Depletion, goblet cells	0/3 (0.0) [0%]	0/3 (0.0) [0%]	0/3 (0.0) [0%]	3/3 (4.0) [100%]	0/3 (0.0) [0%]	0/3 (0.0) [0%]	0/3 (0.0) [0%]	3/3 (1.0) [100%]
Differentiation, squamous	0/3 (0.0) [0%]	0/3 (0.0) [0%]	1/3 (1.0) [33%]	3/3 (1.7) [100%]	0/3 (0.0) [0%]	0/3 (0.0) [0%]	1/3 (1.0) [33%]	1/3 (1.0) [33%]
necrosis	0/3 (0.0) [0%]	0/3 (0.0) [0%]	0/3 (0.0) [0%]	3/3 (1.3) [100%]				
Cyst, intraepithelial					0/3 (0.0) [0%]	0/3 (0.0) [0%]	1/3 (1.0) [33%]	2/3 (1.5) [67%]

Data in the table are presented as follows: number of the ALI cultures affected/number of the ALI cultures examined; (average severity of affected ALI cultures rounded to the nearest tenth of a whole number); (percent incidence rounded to a percentage as a whole number). When applicable, lesions were graded for severity as 1 (minimal), 2 (mild), 3 (moderate), or 4 (marked). NA: not applicable.

## Data Availability

Not applicable.

## Supplementary Materials

The following are available online at https://www.mdpi.com/article/10.3390/ijms23052593/s1.
