# Supplementary material for: Evaluating the Sub-Acute Toxicity of Formaldehyde Fumes in an In Vitro Human Airway Epithelial Tissue Model
Source: Int J Mol Sci. 2022 Feb 26;23(5):2593. doi: 10.3390/ijms23052593 (PMC8910234; doi:10.3390/ijms23052593)
Supplement: Supplementary file 1 [file ijms-23-02593-s001.zip › ijms-1608830-supplementary.pdf]

International Journal of
Molecular Sciences

MDPI

Article

Evaluating the Sub-Acute Toxicity of Formaldehyde Fumes in an In Vitro Human Airway Epithelial Tissue Model

Baiping Ren [1], Qiangen Wu [2], Levan Muskhelishvili [3], Kelly Davis [3], Yiying Wang [1], Diego Rua [4] and Xuefei Cao [1,*]

[1] Division of Genetic and Molecular Toxicology, National Center for Toxicological Research, US Food and Drug Administration, Jefferson, AR 72079, USA; baiping.ren@fda.hhs.gov (B.R.); yiying.wang@fda.hhs.gov (Y.W.)
[2] Division of Biochemical Toxicology, National Center for Toxicological Research, US Food and Drug Administration, Jefferson, AR 72079, USA; qiangen.wu@fda.hhs.gov
[3] Toxicologic Pathology Associates, Jefferson, AR 72079, USA; levan.muskhelishvili@fda.hhs.gov (L.M.); kelly.davis@fda.hhs.gov (K.D.)
[4] Division of Biology, Chemistry, and Materials Science, Office of Science and Engineering Laboratories, Center for Devices and Radiological Health, US Food and Drug Administration, Silver Spring, MD 20993, USA; diego.rua@fda.hhs.gov
* Correspondence: xuefei.cao@fda.hhs.gov; Tel.: +1-(870)543-7723

Citation: Ren, B.; Wu, Q.; Muskhelishvili, L.; Davis, K.; Wang, Y.; Rua, D.; Cao, X. Evaluating the Sub-Acute Toxicity of Formaldehyde Fumes in an In Vitro Human Airway Epithelial Tissue Model. *Int. J. Mol. Sci.* **2022**, *23*, 2593. https://doi.org/10.3390/ijms23052593

Academic Editor: Stefania Marzocco

Received: 7 February 2022
Accepted: 22 February 2022
Published: 26 February 2022

Publisher's Note: MDPI stays neutral with regard to jurisdictional claims in published maps and institutional affiliations.

Copyright: © 2022 by the authors. Licensee MDPI, Basel, Switzerland. This article is an open access article distributed under the terms and conditions of the Creative Commons Attribution (CC BY) license (https://creativecommons.org/licenses/by/4.0/).

Supplementary Materials

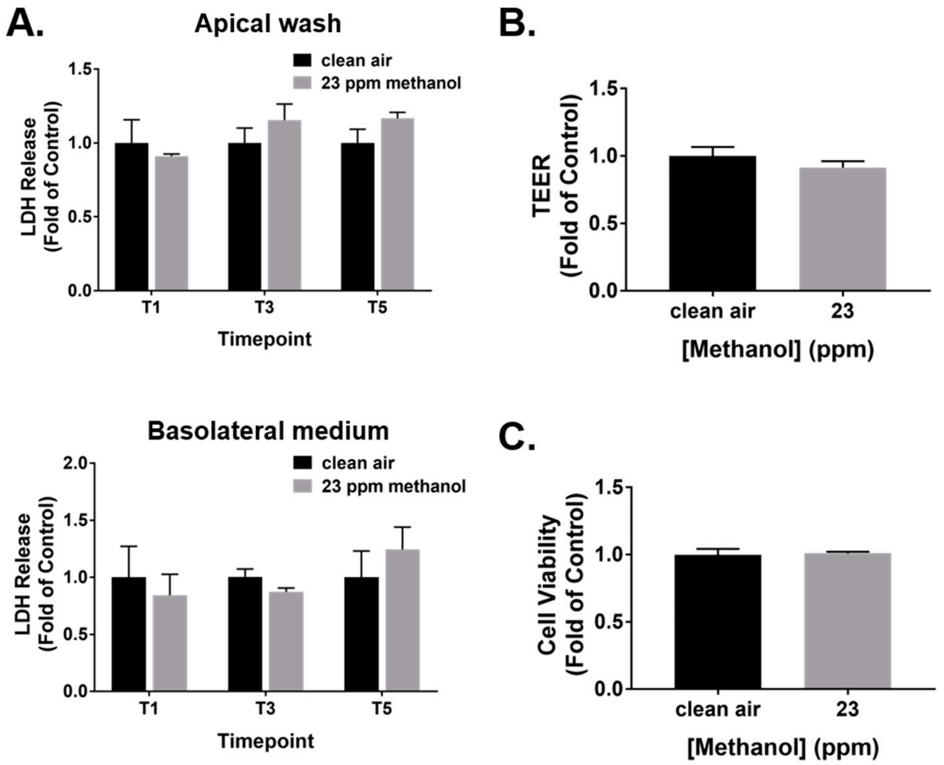

Figure S1. Cytotoxicity of methanol in the ALI cultures. (**A**) Release of LDH into the apical washes and basolateral media induced by methanol was assessed using the LDH assay at T1, T3, and T5. (**B**) Integrity of the ALI airway tissue was measured at T5. (**C**) Cell viability potentially affected by methanol was evaluated using an MTS cell viability assay at T5. Data (n=3) are presented as means ± SEM.

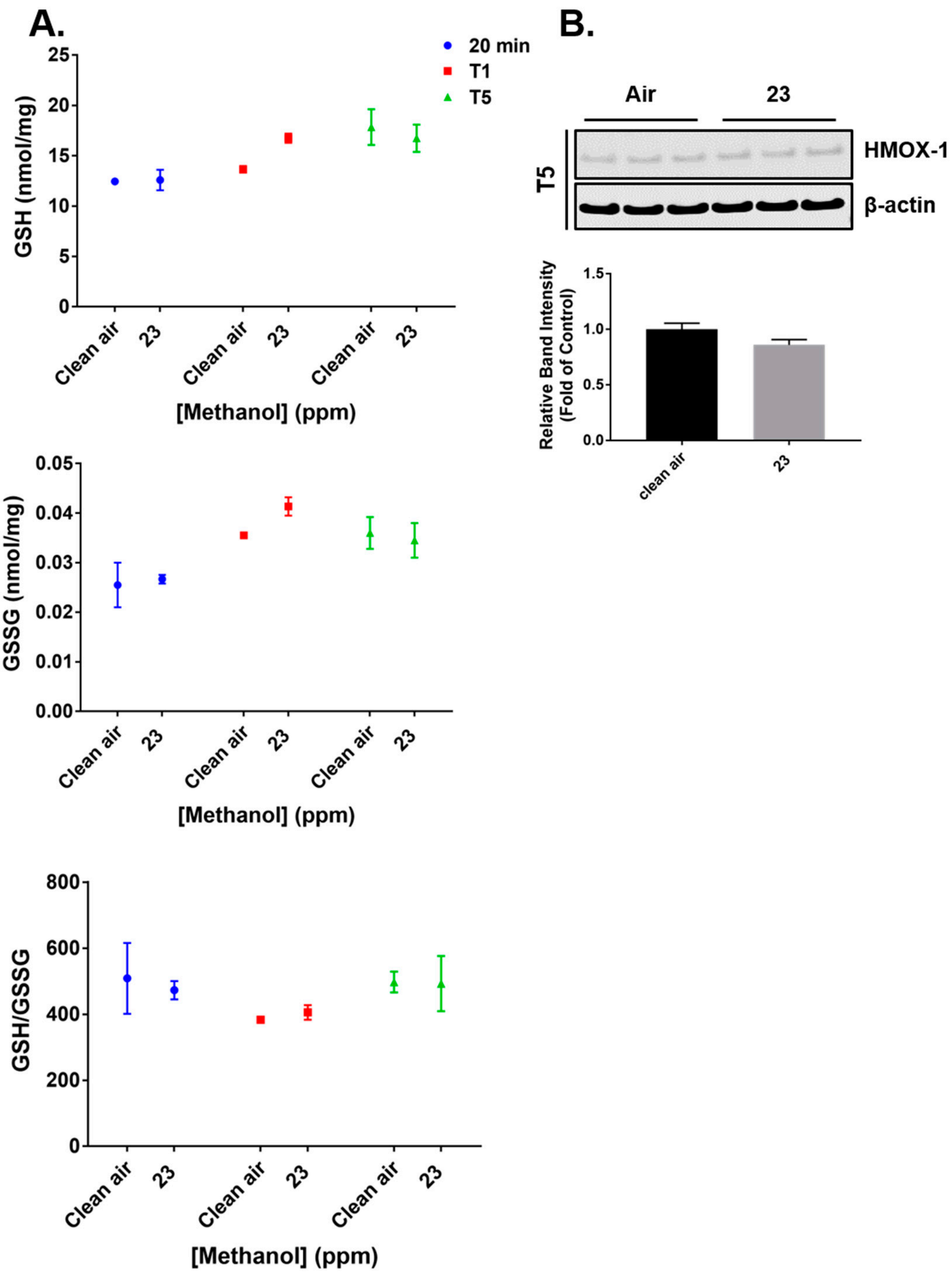

Figure S2. Effect of methanol on oxidative stress. (**A**) Intracellular levels of GSH and GSSG were quantified immediately after the first 4-h treatment (20 min) and at T1 and T5. Ratios of GSH/GSSG were calculated at the respective time points and values normalized to the corresponding clean air-exposed controls. (**B**) Protein expression of HMOX-1 was measured at T5 by immunoblotting. Representative immunoblots are presented (upper panel). Band intensity of HMOX-1 was normalized to that of β-actin (as loading control) for each sample (lower panel). Relative expression of HMOX-1 was expressed as the ratio of the average band intensity of each treatment group to that of the clean air-exposed control. Quantification data (n=3) are presented as means ± SEM.

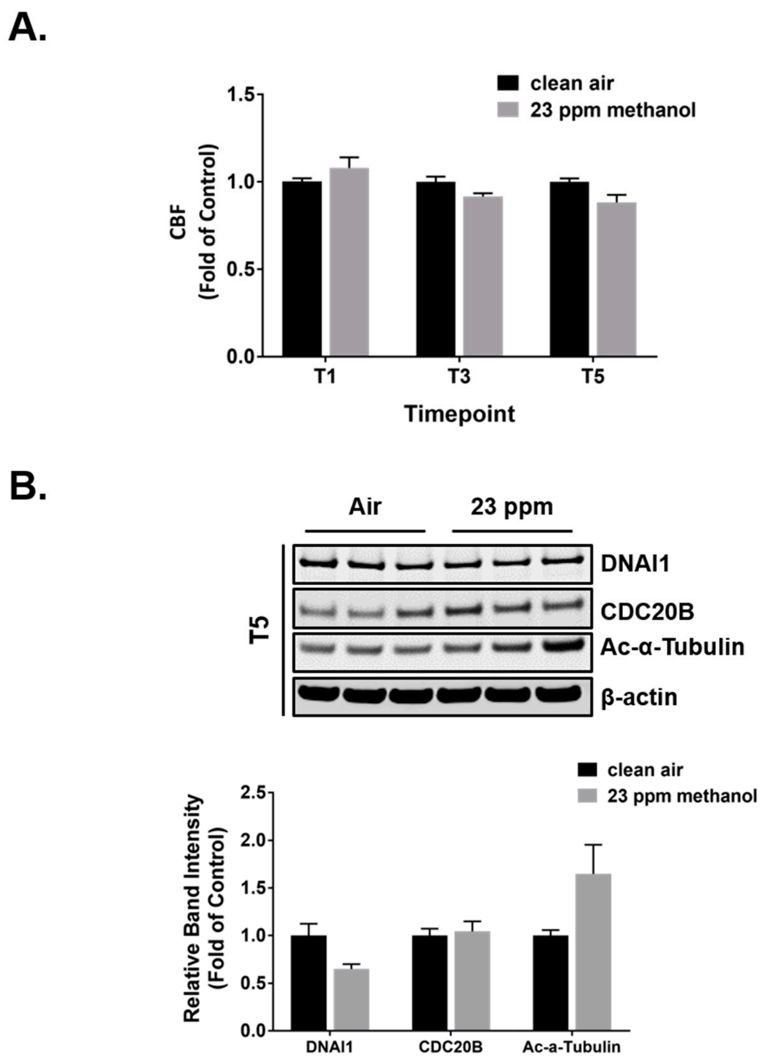

Figure S3. Effect of methanol on ciliary function and structure. (**A**) CBF was measured at T1, T3, and T5. (**B**) Expression of DNAI1, CDC20B, and acetylated-α-tubulin was measured at T5 by immunoblotting. Representative blots are presented. Quantification data (n=3) are expressed as means ± SEM.

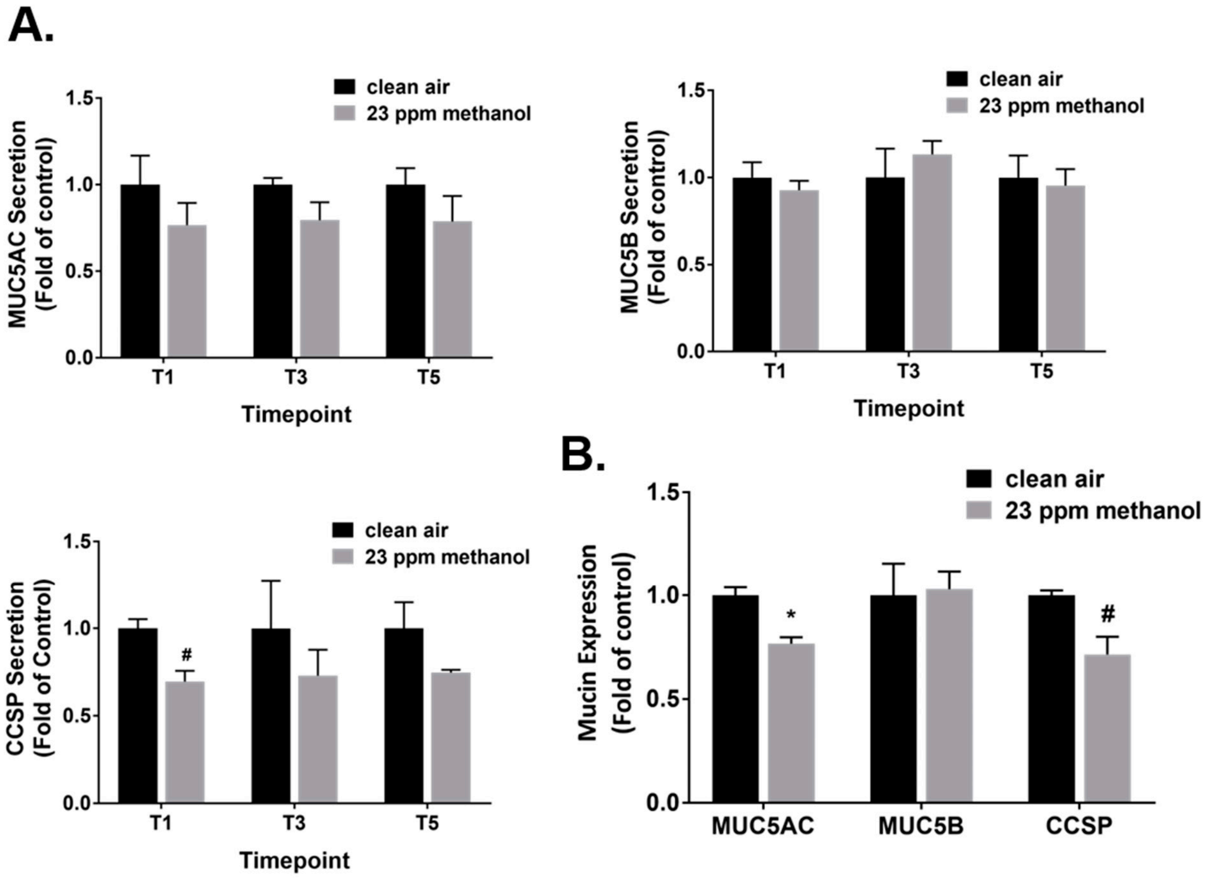

Figure S4. Effect of methanol on mucin homeostasis. (**A**) Secretion of MUC5AC, MUC5B, and CCSP was quantified at T1, T3, and T5 using an ELSA assay. (**B**) Expression of MUC5AC, MUC5B, and CCSP was quantified at T5. Data (n=3) are presented as means ± SEM. $^{\#,*}p < 0.05$ was considered statistically significant compared to the respective clean air-exposed control.

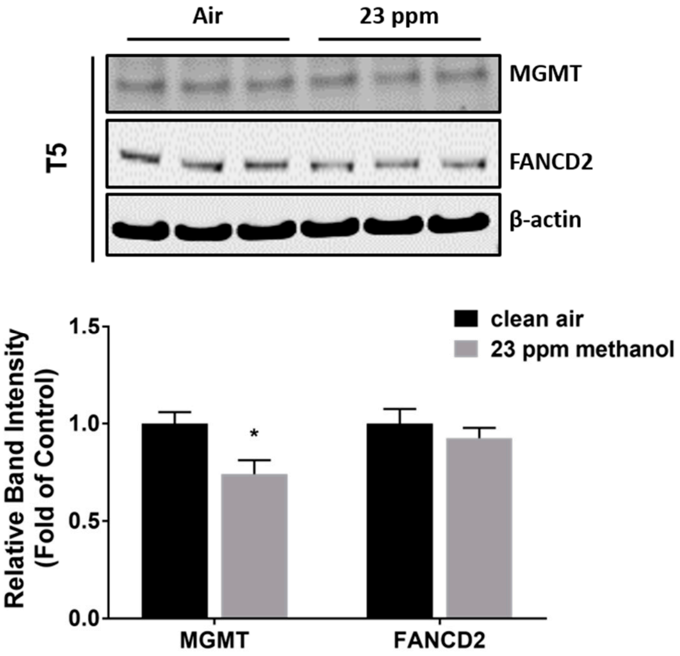

Figure S5. Effect of methanol on DNA repair enzymes. Expression of MGMT and FANCD2 was measured at T5 by immunoblotting. Representative blots were presented in the upper panel. Quantification data (*n*=3) are expressed as means ± SEM. **p* < 0.05 was considered statistically significant compared to the corresponding clean air-exposed control.

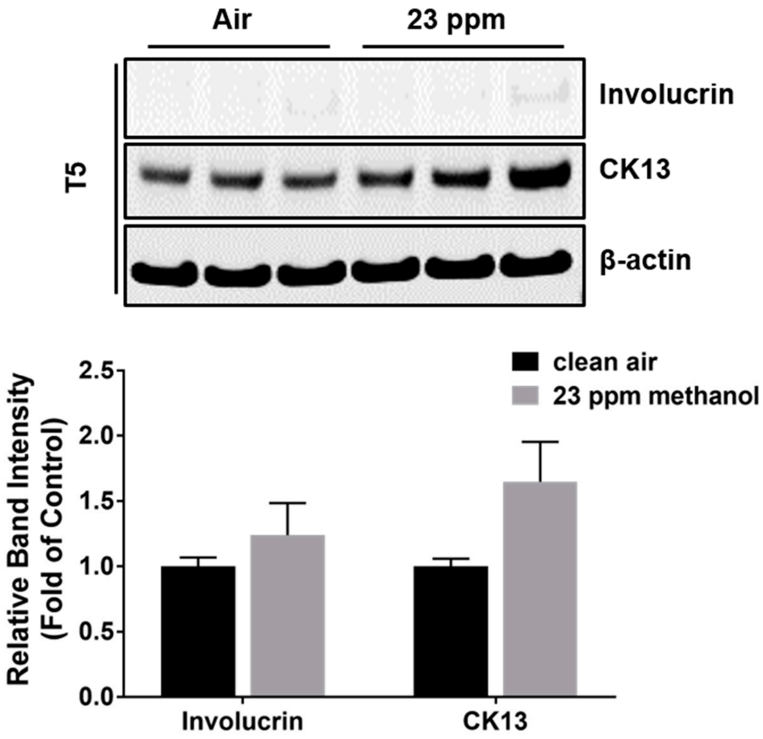

Figure S6. Effect of methanol on squamous differentiation. Expression of involucrin and CK13 was measured at T5 using immunoblotting. Representative blots are presented in the upper panel. Quantification data (*n*=3) are expressed as means ± SEM.

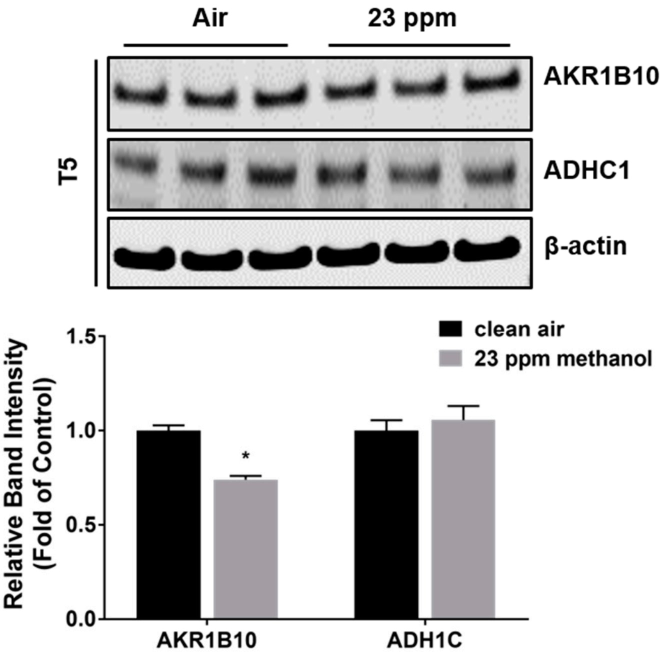

Figure S7. Effect of methanol on AKR1B10 and ADH1C. Expression of AKR1B10 and ADH1C was measured at T5 by immunoblotting. Representative blots were presented in the upper panel. Quantification data (n=3) are expressed as means ± SEM. *$p < 0.05$ was considered statistically significant compared to the corresponding clean air-exposed control.

Table S1. Modulation of inflammatory cytokines by methanol.

Analytes (pg/mL)	T1		T5	
	Clean Air	23 ppm	Clean Air	23 ppm
IL-1β	80.66 (7.30)	70.91 (3.26)	75.40 (3.38)	79.98 (2.45)
IL-2	68.68 (5.98)	55.19 (3.20)	67.44 (4.71)	75.44 (3.16)
IL-8	9814.15 (1993.46)	7125.76 (181.46)	9466.96 (1323.89)	11087.36 (719.38)
TNF-α	98.63 (8.23)	83.37 (5.38)	106.74 (7.10)	115.92 (6.38)
IFN-γ	426.24 (47.45)	353.78 (23.35)	437.57 (45.63)	488.97 (26.81)
GM-SCF	169.99 (11.07)	153.57 (9.24)	182.90 (12.12)	197.44 (9.33)
